# Une association rare de polychondrite atrophiante et de lupus érythémateux systémique: à propos d'un cas

**DOI:** 10.11604/pamj.2025.52.30.47129

**Published:** 2025-09-19

**Authors:** Valentine Séréna Ndong, Ghita Taki, Hicham Harmouche

**Affiliations:** 1 Service de Médecine Interne de l'Hôpital Universitaire Cheikh Zaïd, Université Internationale Abulcasis des Sciences de la Santé, Rabat, Maroc,; 2 Service de Médecine Interne du Centre Hospitalier Universitaire Ibn Sina Rabat, Faculté de Médecine, Université Mohamed V, Rabat, Maroc

**Keywords:** Polychondrite atrophiante, lupus érythémateux systémique, cas clinique, Relapsing polychondritis, systemic lupus erythematosus, case report

## Abstract

L'association de la polychondrite atrophiante chez les patients suivis pour un lupus érythémateux systémique est peu décrite dans la littérature avec une prévalence de 1%. Nous rapportons le premier cas marocain, il s'agit d'une patiente de 44 ans qui a présenté après neuf années d'évolution du lupus, un tableau de polychondrite atrophiante avec une rémission clinique après un suivi de 4 ans sous hydroxychloroquine et une faible dose de prednisone. Dans notre revue de la littérature, nous avons recensé 23 cas de lupus associé à la polychondrite atrophiante. Il s'agit de deux pathologies distinctes dont le mécanisme auto-immun serait impliqué. Ce cas souligne l'importance d'une surveillance mutuelle pour détecter précocement la survenue de l'une ou l'autre pathologie, malgré sa rareté.

## Abstract (en)

The coexistence of relapsing polychondritis in patients under follow-up for systemic lupus erythematosus is rarely reported in the literature, with a prevalence of about 1%. We here report the first Moroccan case, a 44-year-old female patient who, after nine years of lupus, developed relapsing polychondritis and achieved clinical remission after four years of follow-up with hydroxychloroquine and low-dose prednisone. In our literature review, we identified 23 cases of lupus associated with relapsing polychondritis. These are two distinct pathologies in which the autoimmune mechanism is thought to be involved. This case highlights the importance of mutual surveillance to detect early the occurrence of either condition, despite their rarity.

## Introduction

La polychondrite atrophiante (PCA) est une maladie caractérisée par une inflammation récidivante des cartilages de l'oreille, du nez, du larynx et de l'arbre trachéobronchique. Elle est rare et peut parfois être suivie d'une dégénérescence des structures atteintes [1]. Le lupus érythémateux systémique (LES) est une maladie auto-immune caractérisée par la présence d'anticorps antinucléaires dirigés contre les cellules du noyau et les dépôts de complexes immuns responsables des différentes manifestations systémiques [2]. L'association de la PCA chez les patients lupiques est peu décrite dans la littérature avec une prévalence de 1% de la PCA [3]. Nous rapportons le cas d'une patiente présentant une polychondrite atrophiante neuf ans après le diagnostic du lupus avec une revue de la littérature. Ce rapport de cas respecte les directives CARE.

## Patient et observation

**Information relative à la patiente:** une femme de 44 ans, née au Maroc, allergique à la pénicilline, était suivie depuis 13 ans pour un LES.

**Résultats cliniques:** elle avait une polyarthrite des poignets et des coudes, un rash malaire, une biopsie cutanée faite révélant une dermite d'interface compatible avec un lupus. Sur le plan biologique, elle avait un syndrome inflammatoire biologique avec une vitesse de sédimentation accélérée à 56mm, une CRP négative, le complément C3 à 0.76g/l, un C4 à 0,16g/l, les anticorps antinucléaires étaient positifs à 1/2560 avec fluorescence homogène, les anti-DNA positifs et les anti-ECT négatifs. Les anti-CCP et le FR étaient négatifs. Elle n'avait pas d'atteinte rénale, la protéinurie de 24 heures négative et la créatinine à 6,3mg/l. La patiente n´avait pas de sérite, pas d'atteinte neuropsychiatrique, ni d'atteinte hématologique et la biologie anti-phospholipide était négative. Elle était traitée par de l'hydroxychloroquine à la dose de 6,5 mg/kg/jour au long cours et 10mg par jour de prednisone arrêtée après diminution progressive.

**Figure 1:**
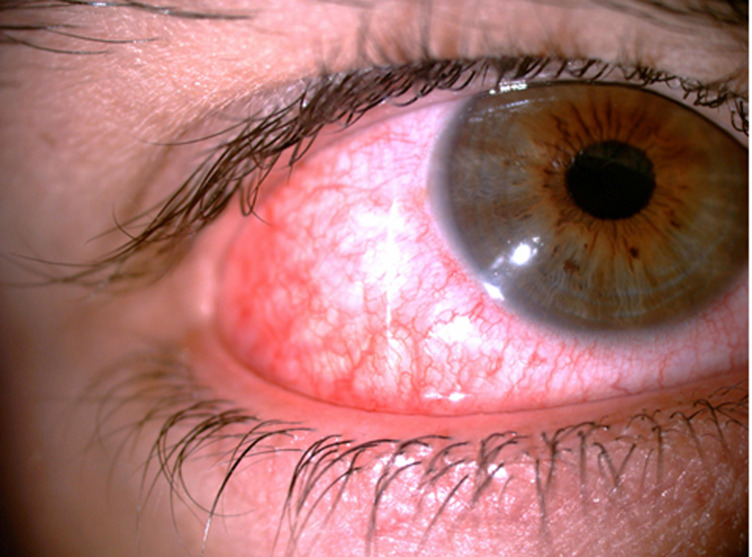
sclérite de l'œil droit à l'examen de la lampe à fente chez notre patiente

**Figure 2:**
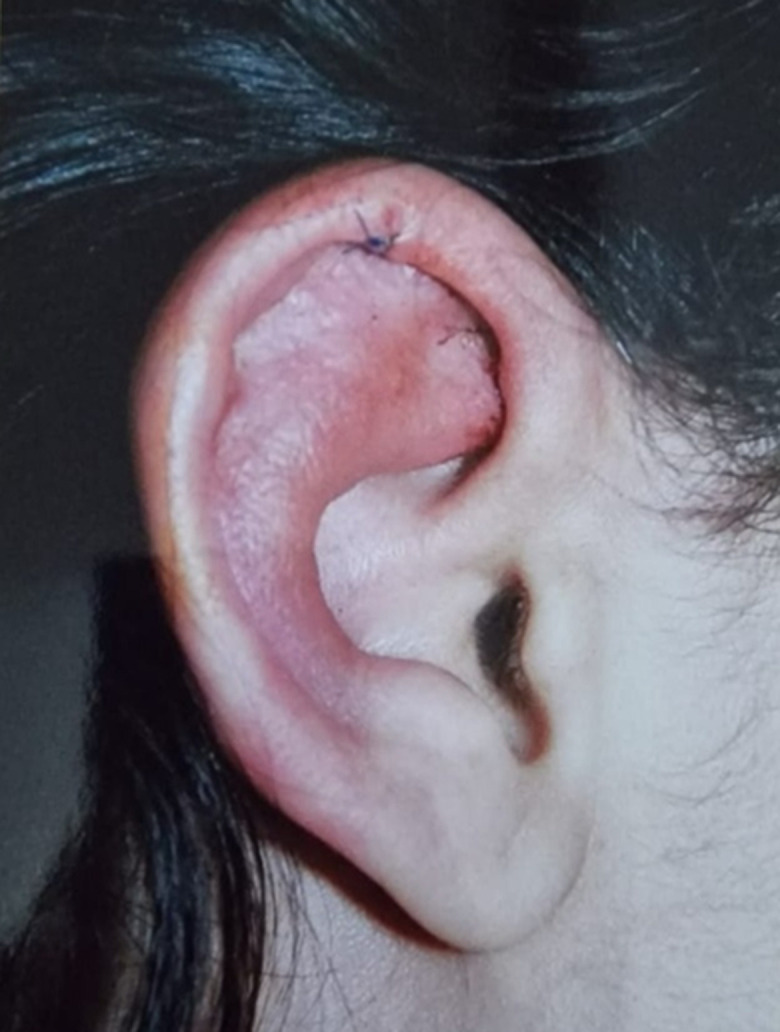
chondrite de l'oreille droite épargnant le lobule chez notre patiente

**Chronologie:** neuf ans après ce diagnostic, la patiente avait présenté une rougeur et une douleur oculaire bilatérale secondaire à une sclérite (Figure 1). Elle a présenté des polyarthralgies associées à des épisodes de chondrite des oreilles épargnant le lobule (Figure 2), dont la biopsie a montré une absence de granulome et un aspect compatible avec une PCA.

**Démarche diagnostique:** elle n´avait pas d´atteintes nasale, laryngo-trachéale ni cochléo-vestibulaire. Sur le plan biologique le dosage des anticorps anticytoplasme des polynucléaires neutrophiles (ANCA) était négatif et l'enzyme de conversion de l'angiotensine (ECA) était normale. Les TDM ORL et thoracique n'objectivaient pas de sinusite, pas d'atteinte trachéale.

**Intervention thérapeutique:** le diagnostic de PCA avait été posé et ces poussées avaient été contrôlées par de la prednisone 0,5mg/kg/jour, soit 30mg par jour, avec décroissance progressive.

**Suivi et résultats:** actuellement, après un suivi de 4 ans, on ne note pas de récidive de poussée oculaire et cartilagineuse de la PCA; la patiente est en rémission du LES sous hydroxychloroquine à la dose de 400mg/jour.

**Perspective et consentement éclairé du patient:** la patiente est actuellement en rémission et a donné son consentement éclairé pour la publication de cet article.

## Discussion

La fréquence de la PCA associée au LES est peu décrite dans la littérature. Kitridou *et al*. rapportaient chez les patients lupiques une prévalence de 1% de la PCA [3]. Small *et al*. suggéraient à travers 2 cas de la littérature que la polychondrite devrait être considérée comme une présentation atypique du LES même si les poussées de chondrite n'étaient pas associées aux poussées typiques lupiques [4]. En 1994, Harisdangkul *et al*. avaient retrouvé à l’issue d’une revue de la littérature 16 cas de cette association dont le mécanisme physiopathologique sous-jacent est encore mal connu [5]. Le lien entre ces deux maladies pourrait s'expliquer par le lit de l'auto-immunité. L'origine auto-immune de la PCA décrite sur le plan physiopathologique résulterait de l'action des anticorps contre certains antigènes du cartilage encore inconnus. L'auto-immunité est suggérée par la présence du HLA DR4 présent dans 50% des cas; son association aux maladies auto-immunes dans 30% des cas; la présence d'anticorps anticollagène de type II dans 30% des cas et la présence de lymphocytes T CD4+, plasmocytes et de dépôts immuns dans les lésions de chondrite [1]. Notre patiente a une PCA dont le diagnostic répond aux critères de classification établis par Michet en 1986, qui demeurent pertinents et sont mentionnés par Puéchal *et al*. dans leurs travaux [1] et aux critères de classification du LES de l’ACR 1997, au moment du diagnostic, dont la mise à jour est faite en 2019 [2].

À ce jour, vingt-trois cas ont été décrits dans la littérature. Les caractéristiques de ces cas sont présentées dans le Tableau 1 [5-9], les 17 premiers ayant été décrits précédemment par Harisdangkul *et al*. [5]. La chronologie de survenue de la PCA chez les patients qui ont un LES n'est pas bien définie dans la littérature. Ce délai est long chez notre patiente, la PCA survenant après 9 années d'évolution en dehors d'une poussée lupique. Dans leur revue de la littérature, Harisdangkul *et al*. mentionnent les travaux de Marshall et de Small, qui rapportent également une longue durée d'évolution vers la rémission du LES, respectivement de 6 ans et 8 ans pour les cas 1 et 5. Concernant les autres cas, c'est la PCA qui a permis de mettre en évidence un LES associé à des anticorps spécifiques [5].

**Tableau 1:** association d'une polychondrite chronique atrophiante et d'un lupus érythémateux systémique chez 23 patients et notre cas

Cas numéro	Auteurs	Age	Sexe	Race	Caractéristiques du LES	Caractéristiques de la PCA	Traitement
1	*Marshall J, 1964	41	M	Blanche	Arthrite, myalgie, cellule LE positive	Chondrite bilatérale des oreilles 6 ans après le début de l'arthrite	-
2	*Kaye RL, 1964	53	F	Inconnu	Tableau clinique évocateur du LES et positivité de la cellule LE	Chondrite oreille, déformation nasale, arthrite, atteinte laryngée, costale et inflammation oculaire	Prednisone, hydroxychloroquine
3	*Cody TDR, 1971	55	F	Inconnu	Biopsie rénale montrant une glomérulonéphrite subaiguë	Polyarthrite inflammatoire, chondrite et atteinte audiovestibulaire	Prednisone
4	*Rogers PH, 1973	60	F	Noire	Cellules LE positives, AAN positifs, fausse sérologie syphilitique positive	Chondrite du nez, des oreilles, atteinte audiovestibulaire, érythème noueux, polyarthralgie, conjonctivite, Ac anti cartilage	Prednisone
5	Small P, 1980	29	F	Blanche	Glomérulonéphrite membranoproliferative, photosensibilité, atteinte SNC, arthrite, AAN homogène et cellule LE positive	Chondrite récidivante des 2 oreilles 8 ans après le diagnostic du LES	Corticoïde, immunosuppresseur
6	Small P, 1980	34	F	Blanche	Lupus discoïde, arthralgie récidivante, AAN homogène, présence de cellule LE	Chondrite récurrente oreille droire et diminution du complément dans le liquide périchondral	Traitement local pour lupus, résolution spontanée de la chondrite
7	*Job Deslandre, 1983	26	F	Blanche	Rash malaire, polyarthrite, AAN et cellule LE positives, diminution du complément	Déformation nasale et chondrite	-
8-10	*Kitridou R, 1984	Inconnu	Inconnu	Inconnu	Tous les 3, LES, cryoglobulinémies, DNA positifs	Chondrite récidivante, biopsie cartilage avec dépôt d'IgG et C3, Ac anti collagène II	-
11-16	*Isaak BL, 1986	Inconnu	Inconnu	Inconnu	6/112 patients avec PCA sans description clinique	Pas de description clinique	-
17	Harisdangkul V, 1994 [5]	38	F	Blanche	CH50 bas, VS accélérée, AAN, DNA positifs	Chondrite auriculaire récidivante, polyarthrite, épisclérite	Prednisone
18-20	Piette JC, 1997 [6]	Inconnu	F	Inconnu	3/180 SAPL et LES	Pas de description clinique	-
21	Lenormand C, 2008 [7]	31	M	Blanche	Photosensibilité, lésion cutanée chronique lupique, FAN positifs sans spécificité, C4 et CH50 bas	Polyarthralgie, chondrite auriculaire récidivante	Dapsone, hydroxchloroquine, photoprotection
22	Bellon N, 2013 [8]	32	F	Inconnu	Microangiopathie thrombotique avec ADAMS 13 bas et présence Ac anti ADAMS 13, compliqué d'AVCI sylvien gauche, AAN et DNA positifs	Chondrite des oreilles et du nez	Hydroxycholoroquine, corticoïde, échange plasmatique, Rituximab, relais Azathioprine
23	Nguyen MA, 2016 [9]	47	F	Inconnu	Photosensibilité, ulcérations buccales, néphrite lupique, FAN et anti Sm positifs	Chondrite auriculaire bilatérale	Prednisone, hydroxychloroquine
24	Notre cas	44	F	Marocaine	Polyarthrite, rash malaire, AAN et anti DNA positifs	Chondrite auriculaire récidivante, polyarthralgies, sclérite	Hydroxychloroquine, prednisone

* Ces études ont été mentionnées par Harisdangkul *et al*. [5].

Le traitement de la PCA est individuel, fonction de l'atteinte clinique, mais associe globalement les anti-inflammatoires, la colchicine, la dapsone et surtout la corticothérapie à faible dose. Les principaux facteurs de mauvais pronostics sont l’atteinte respiratoire, l’association au syndrome myélodysplasique et les infections secondaires au traitement [1]. Le traitement du LES repose sur l'hydroxychloroquine, les corticoïdes, les immunosuppresseurs en fonction de l'atteinte d'organe dont les atteintes rénale, neuropsychiatrique et hématologique conditionnent le pronostic [10]. Notre patiente a été traitée par hydroxychloroquine et prednisone comme la majorité des patients et selon les recommandations adaptées et/ou en fonction de l'organe atteint et du degré de sévérité.

L'évolution de notre patiente était favorable au diagnostic après 4 ans de suivi, cependant des associations plus graves ont été décrites comme celles de Bellon *et al*. Ils avaient décrit le cas d'une patiente de 32 ans avec une microangiopathie thrombotique compliquée d´AVCI sylvien gauche révélant le LES associée à une PCA avec chondrite des oreilles et du nez. Sur le plan biologique elle avait une anémie à 11g/dL, une thrombopénie à 27 Giga/L), la présence de 6% de schizocytes, une haptoglobine indétectable, un taux de lactate déshydrogénase (LDH) élevé et un test de Coombs négatif, la recherche des anti-phospholipides était négatif, un taux d´ADAMS 13 bas, présence d´Ac anti ADAMS 13, les compléments C3 et C4 consommés, des FAN positifs à 1/1280 avec des Ac anti DNA, SSA et SSB positifs, une PCA avec chondrite du nez et des oreilles. Une amélioration a été notée après bolus de corticoïdes, échanges plasmatiques et traitement par rituximab [8]. Nous avons aussi les trois cas rapportés par Piette *et al*. ayant un syndrome des anti-phospholipides associé au LES - PCA [6].

## Conclusion

Nous avons rapporté le premier cas marocain présentant une association rare entre LES et PCA. Même si l´on ne sait pas encore ce qui constitue l´élément déclencheur de cette association ni quelle est la pathologie première qui fait le lit de la deuxième, cela souligne l´importance d´une surveillance réciproque pour détecter précocement la survenue de l'une ou l´autre de ces deux affections auto-immunes malgré leur rareté.
